# The Institutional Worker

**Published:** 1911-10-21

**Authors:** 


### The INSTITUTIONAL WORKER

**Being a Special Supplement to "The Hospital"**

### Editor's Notices.

**Contributions:**

Contributions should be written, or preferably typed, on one side of the paper only, and all articles sent in are accepted upon the distinct understanding that they are forwarded to The Hospital only.

The Editor cannot undertake to return MSS. not used, but when a stamped directed envelope is enclosed the MSS. may be returned if a special request is made.

Accepted articles and paragraphs of news will be paid for after publication at the scale rate.

**Address:**

To prevent delay all contributions and letters on editorial business must be addressed exclusively to the Editor, "The Hospital," 29 Southampton Street, Strand, London, W.C. It is important that this regulation shall be strictly observed.

**Correspondence:**

Correspondence on all subjects is invited. The name and address of correspondents must be given as a guarantee of good faith, but not necessarily for publication.

**Special Articles:**

Special articles are invited, and questions, inquiries, and paragraphs upon all matters relating to the work, administration, and management of general, special, mental, fever, cottage and convalescent hospitals, sanatoria, homes, institutions, societies and organisations for the treatment or care of the sick, injured and dependents of all classes. Every matter affecting the interests and welfare of the staff of all grades working in these institutions will receive special consideration.

**Administrative Medicine, Research and Items of News:**

Special terms will be paid for approved articles on subjects in this wide field by experts who have made some section of it their special study and interest. We wish to give prominence to every movement tending to advance accuracy of diagnosis, efficiency in treatment, and the development of modern methods to eradicate disease and promote the welfare of the suffering.

**A Bureau of Information:**

We invite inquiries and applications for information and help in providing for that numerous class of sufferers who require special care or house-room which they cannot obtain in their own homes or secure for themselves. **We cannot, however, prescribe or recommend practitioners.**

**Books for Review:**

Publishers are particularly requested to send advance proofs of any new books of importance whenever possible, as the Editor has made arrangements to publish immediate reviews on a new plan.

**Photographs, Plans, Blocks and Illustrations:**

It is requested that wherever possible MSS. may be accompanied by illustrations in any of the above forms. The name of the sender and of the article to which it belongs should be written on the back of each photograph, drawing, or block for purposes of identification.

**THE HOSPITAL DISPENSER:**

The Editor begs to thank those who have sent him suggestions. He wishes to help every worker employed in an institution and to further their interests. This week the position of dispensers engaged in institutions has formed a prominent feature of the correspondence, and will be made one of the new departures of The Hospital. The Editor hopes that other groups of institutional workers will make their work difficulties and needs known, so that they may receive adequate treatment in The Hospital in due course. It is only by co-operation through correspondence and interviews that the interests of all workers can be adequately promoted. Everyone who has a suggestion or even a grievance should send it for consideration on its merits.

**Local Papers:**

Newspapers containing reports or news paragraphs **should be marked** and addressed to the Sub-Editor.

### Manager's Notices.

Letters relating to the publication, sale, and advertising departments of The Hospital must be addressed to the Manager, "The Hospital," The Hospital Building, 28 and 29 Southampton Street, Strand, London, W.C.

**Circulation and Sale:**

The Hospital is on sale at all newsagents and bookstalls every Friday, price one penny. The rates of subscriptions, post free from The Hospital office, have been reduced and are now as follows:—

| | United Kingdom s. d. | The Colonies and Abroad s. d. |
|---|---|---|
| For one year ... ... ... | 6 6 | 8 8 |
| ,, six months ... ... | 4 0 | 5 0 |
| ,, three months ... ... | 2 0 | 2 6 |

**Subscriptions**, which may begin at any time, are payable in advance. Cheques and Post-Office orders, crossed London County and Westminster Bank, Covent Garden branch, should be made payable to the Manager of The Hospital and addressed to him at The Hospital Building, 28 and 29 Southampton Street, Strand, London, W.C.

**Advertisements:**

All advertisements for The Hospital must reach the Manager not later than Wednesday morning in each week. For scale of charges see pages v and 2.

**Special Rates** will be quoted for those institutions which agree to send all their vacancy, contract, and public notices to The Hospital each year, so as to make it a file of such announcements for ready reference.

**Classified Advertisements:**

All small advertisements will be classified, for this section of the paper is widely read and of special interest. We wish to encourage those wanting appointments who are attracted to institutional work, and therefore offer them the reduced rate of *twenty-one words and under for* 1s., and *for every ten and part of ten words beyond*, 6d.

**The Staff and the Hospital:**

The Editor has in preparation some interesting articles on types of hospital workers, the nature of the work which devolves upon each section of the staff, and the qualifications which must contribute to success in public institutions. The Hospital is the only newspaper appealing to institutional workers of all grades, and with their co-operation everything will be done to make it additionally instructive, attractive, and useful each week.

**The Coupon System:**

A coupon will be found at the bottom of the third inside page of the cover each week. This coupon must be attached to every question or inquiry to which an answer is desired in The Hospital.

### APPOINTMENTS VACANT.

### Poor-Law Vacancies.

### Appointments Vacant.

Assistant Matron (£40), Southampton Union. Mr. A. J. Walden, C. to G., Southampton. Oct. 26.

Assistant Midwifery Nurse (£30), Kensington Guardians. Matron of the Infirmary, Marloes Road, Kensington.

Assistant Nurse (£30), St. Ives (Hunts.) Union. G. D. Day, C. to G., St. Ives, Hunts. Oct. 23.

Assistant Nurse (£16), Stepney Union. T. G. Stacey, C. to G., Barnes Street, Stepney, E. Oct. 25.

Assistant Nurse (£28), Bromley Union. E. Haslehurst, C. to G., Union Offices, Park House, Bromley, Kent. Oct. 23.

Assistant Nurse (£25), Parish of Liverpool. G. W. Coster, Vestry Clerk, Parish Offices, Liverpool. Oct. 21.

Assistant Nurse (£22), Malling Union. Mr. F. J. Allinson, C. to G., West Malling. Oct. 23.

Assistant Nurse (£26), Minehead and Williton (West) District Hospital Committee. T. H. Hosegood, Clerk to the Committee, Minehead. Oct. 23.

Assistant Nurse (£26), Lincoln Union. W. B. Danby, C. to G., Workhouse, Lincoln. Oct. 30.

Assistant Nurse (£30), West London School District. F. G. Beeching, Clerk West London Schools, Ashford, Middlesex. Oct. 30.

Assistant Nurses (£36), Grimsby Union. J. F. Wintringham, C. to G., St. Mary's Chambers, Grimsby. Oct. 23.

Assistant Nurses (£30), Llanelly Union. Mr. D. C. Edwards, C. to G., Llanelly. Oct. 23.

Assistant Superintendent Nurse (£35). T. G. Stacey, C. to G., Barnes Street, Stepney, E. Oct. 25.

Attendant (£28), Mile End Guardians. B. Catmur, C. to G., Bancroft Road, Mile End, E. Oct. 26.

Charge Nurse (£30), Steyning Union. A. Flowers, C. to G., Shoreham-by-Sea. Oct. 24.

Charge Nurses (£36), Grimsby Union. J. F. Wintringham, C. to G., St. Mary's Chambers, Grimsby. Oct. 23.

Charge Nurse (£40), West Derby Union. C. to G., Union Offices, Brougham Terrace, Liverpool. Oct. 27.

Charge Nurse (£30), Staines Union. F. Hutchinson, C. to G., Stanwell Road, Ashford, Middlesex. Oct. 24.

Charge Nurse (£30), West Bromwich Guardians. A. H. Ward, C. to G., 22 Lombard Street, West Bromwich. Oct. 25.

Charge Nurse (£30), South Shields Union. J. W. Coulson, C. to G., Barrington Street, South Shields. Oct. 26.

Female Attendant (£25), York Union. G. Sykes, C. to G., York. Oct. 24.

Female Attendants (£25), West Ham Union. T. Smith, C. to G., Union Road, Leytonstone. Oct. 25.

Female Head Attendant (£35), Bristol Guardians. J. J. Simpson, C. to G., Bristol. Oct. 21.

Female Lunatic Ward Nurse (£20), Southwark Union. S. Wood, C. to G., Ufford Street, Blackfriars, S.E. Oct. 24.

Female Night Attendant (£23), Ashton-under-Lyne Union. G. H. Partington, C. to G., Ashton-under-Lyne. Oct. 24.

Foster-Mothers (2) (£30), Reigate Guardians. F. C. Morrison, C. to G., Reigate. Oct. 25.

Head Nurse (£30), Liskeard Union. A. de C. Glubb, C. to G., Liskeard. Oct. 21.

Head Nurse (£30), Holbeach Union. S. S. Mossop, Jun., C. to G., Holbeach. Oct. 28.

House-Mother (£20), Romford Union. C. Bloomfield, C. to G., Romford. Oct. 23.

Junior Clerk (£25), Hackney Union. F. R. Coles, C. to G., Homerton, N.E. Oct. 23.

Junior Assistant Nurse (£12), Orsett Union. C. Asplin, C. to G., Grays. Oct. 25.

Male Attendant (£30), York Union. G. Sykes, C. to G., York. Oct. 24.

Male Imbecile Attendant (£30), Burnley Union. J. S. Horn, C. to G., Burnley. Oct. 25.

Master and Matron (£50 and £40), Thornbury Union. H. P. Thurston, C. to G., Thornbury, Gloucester. Oct. 26.

Medical Superintendent's Clerk (£45), Fulham Guardians. E. J. Mott, C. to G., 129 Fulham Palace Road, W. Oct. 24.

Night Nurse (£25), Biggleswade Union. G. Wagg, C. to G., Biggleswade. Oct. 21.

Nurse (£35), Freebridge Lynn Union. Mr. W. Cross, C. to G., King's Lynn. Oct. 26.

Nurse (£25), Worksop Union. J. S. Whall, C. to G., 66 Bridge Street, Worksop. Oct. 24.

Nurse (£30), Hemel Hempstead Union. L. Smeathman, C. to G., 1 The Broadway, Hemel Hempstead. Oct. 23.

Nurse (£35), Junior Assistant Nurse (£14), Basingstoke Union. H. W. Chandler, C. to G., Basingstoke. Oct. 23.

Nurse (£36), Ipswich Guardians. L. W. Greenhalgh, C. to G., Ipswich.

Nurses (2) (£30 each), Winchester Guardians. Mr. F. Faithfull, C. to G., Winchester. Oct. 30.

Probationer Nurse (£15), Christchurch Union. A. Druitt, C. to G., Christchurch. Oct. 21.

Probationer Nurse (£12), Uxbridge Union. C. Woodbridge, C. to G., 38 High Street, Uxbridge. Oct. 28.

Relieving Officer, Etc. (£102 and commission), Erpingham Union. J. T. Willis, Deputy C. to G., Cromer Road, Sheringham. Oct. 28.

Relieving Officer, Vaccination Officer, and Collector (£71, commission and fees), Long Ashton Union. A. E. Hicks, C. to G., Flax Bourton. Oct. 23.

Staff Nurse (£26), St. Pancras Guardians. J. E. P. Hall, C. to G., Town Hall, Pancras Road, N.W.

Storekeeper and Assistant Clerk (£40), Merthyr Tydfil Union. Mr. F. T. James, C. to G., Merthyr Tydfil. Oct. 26.

Superintendent Nurse (38), Malling Union. Mr. F. J. Allinson, C. to G., West Malling. Oct. 23.

Valuer, Blean Union. Mr. J. E. Burch, C. to G., 39 Castle Street, Canterbury. Oct. 30.

Ward Sister (£32), Fulham Guardians. E. J. Mott, C. to G., 129 Fulham Palace Road, W. Oct. 25.

Ward Sister (£30), Southampton Union. A. J. Walden, C. to G., Workhouse, Southampton. Oct. 28.

Ward Sister (£32), Prestwich Union. E. W. Ogden, C. to G., Cheetham Hill Road, Manchester. Oct. 30.

Ward Sister (£35), Bradford Union. G. M. Crowther, C. to G., 22 Manor Row, Bradford. Oct. 24.

Ward Sister (£30), St. Marylebone Infirmary. Apply Matron.

### Situations Vacant.

Barber and Bath Attendant (£30), Bromley Union. E. Haslehurst, C. to G., Bromley, Kent. Oct. 23.

Barber and General Assistant (£25), Long Ashton Union. A. E. Hicks, C. to G., Flax Bourton. Oct. 23.

Dispensary Porter (9s. weekly), Hammersmith Guardians. The C. to G., 206 Goldhawk Road, Shepherd's Bush, W. Oct. 30.

Female Cook (£35), Southwark Union. S. Wood, C. to G., Ufford Street, Blackfriars, S.E. Oct. 24.

Female Cook (£20), Bedwellty Union. H. J. C. Shepard, C. to G., Tredegar. Oct. 24.

Female General Assistant (£20), Watford Union. H. M. Turner, C. to G., Bristol. Oct. 21.

Male Cook (£35), Bermondsey Guardians. E. Pitts Fenton, C. to G., 283 Tooley Street, S.E. Oct. 26.

Porter (£25), Swindon and Highworth Union. J. P. Kirby, C. to G., Swindon. Oct. 24.

Porter, Etc. (£25), Biggleswade Union. G. Wagg, C. to G., Biggleswade. Oct. 21.

Portress and Laundry Superintendent (£20), Runcorn Union. G. F. Ashton, C. to G., Runcorn. Oct. 23.

### Institutional and Administrative Appointments.

Lady Housekeeper (£40), Cumberland Infirmary, Carlisle. J. G. Howitt, Secretary. Oct. 21.

Matron (£70), Hospital for Women, Nottingham. Apply Secretary. Oct. 31.

Private Nurses (£40), Norfolk and Norwich Hospital. Apply Matron. Oct. 25.

Staff Nurse (28), Colwyn Bay Cottage Hospital. Apply Hon. Secretary. Oct. 23.

Staff Nurse (£24), Bangor (County Down) Infirmary. Apply Matron. Nov. 1.

### Municipal Vacancies.

### Appointments Vacant.

Accountant Clerk (£156), Southampton C.C. Mr. W. J. Taylor, County Surveyor, Winchester. Oct. 23.

Borough Treasurer and Accountant (£500), Sunderland T.C. Mr. F. M. Bowey, Town Clerk, Sunderland. Oct. 31.

Cemetery Superintendent (£130), Battersea B.C. W. M. Wilkins, Town Clerk, Battersea. Oct. 27.

Chief Architectural Assistant (Education Department) (£200), Chief Architectural Assistant for General County Work (£200), Junior Architectural Assistant (Education Department) (£130), Junior Architectural Assistant for General County Work (£130), Essex C.C. J. H. Goold, Clerk to the Council, Chelmsford. Oct. 28.

Chief Inspector (Waterworks) (£3), Sub-Inspector (£2 10s.), Burnley T.C. P. Thomas, Town Clerk, Burnley. Oct. 21.

Clerk (£200), Cornwall County Asylum. Mr. M. F. Edyvean, Clerk to the Visitors, Bodmin. Oct. 21.

Clerk of the Peace (£755), Pembroke Standing Joint Committee. M. George, Shire Hall, Haverfordwest. Nov. 3.

Deputy Borough Engineer (£180), St. Helens T.C. W. H. Andrew, Town Clerk, St. Helens. Oct. 23.

Female Health Visitor (£80), Barking Urban District Council. H. Hargreaves, Clerk, Public Offices, Barking. Oct. 24.

Gas Engineer and Manager (£1,000), Nottingham T.C. J. A. H. Green, Town Clerk, Nottingham. Nov. 2.

Health Visitor (£80), Kirkby-in-Ashfield U.D. Council. S. B. Hibbert, Clerk, 45 Westgate, Mansfield, Notts. Oct. 28.

Health Visitor (£105), Borough of Bermondsey, F. Ryall, Town Clerk, Bermondsey. Oct. 31.

Inspector of Nuisances (£100), Bury St. Edmunds T.C. A. P. Wheeler, Town Clerk, Bury St. Edmunds. Oct. 25.

Inspector of Nuisances, Etc. (£150), Thornbury R.D.C. H. P. Thurston, Clerk to the Council, Thornbury, Gloucester. Oct. 26.

Matron (£80), Brighton County Borough Asylum. H. Talbot, Town Hall, Brighton. Nov. 6.

Probationer (£16), Sittingbourne and Milton Joint Hospital Board. Matron, Keycol Hill, Sittingbourne.

Probationer Nurse (£16), Llandaff and Dinas Powis R.D. Council. M. Warren, Clerk, 20 Park Place, Cardiff. Oct. 26.

School Nurse Attendant (£60), Birmingham Education Committee. J. A. Palmer, Secretary for Education, Edmund Street, Birmingham.

School Nurses (£75), Swansea Education Committee, A. W. Halden, Clerk, Education Offices, 9 Grove Place, Swansea. Nov. 1.

Staff Nurse (£30), Barry U.D. Council. T. B. Tordoff, Clerk, Council Offices, Barry, Glam. Oct. 25.

Superintendent of Cemetery (£75), Watford Burial Board. T. J. Broad, Clerk to the Board, Watford. Oct. 31.

### Situations Vacant.

Town Hall Keeper (£26 and residence), Wokingham T.C. Mr. J. May, Town Clerk, Wokingham. Oct. 28.

### Miscellaneous Vacancies.

### Situations Vacant.

Assistant Laundress (£20), Monyhull Colony for Epileptics. Matron of the Colony, King's Heath, Birmingham.

Gardener (26s. weekly), Salop County Asylum. Medical Superintendent of the Asylum, Shrewsbury.

Head Laundress (£30), Berry Wood Asylum, Northampton. Apply Medical Superintendent.

Head Laundress (£32), Berks Asylum. Medical Superintendent of the Asylum, Wallingford.

